# Routine 3D SSFP cine imaging for improved analysis of myocardial volumetry and deformation

**DOI:** 10.1186/1532-429X-18-S1-P330

**Published:** 2016-01-27

**Authors:** Bram Ruijsink, María N Velasco Forte, Miguel S Vieira, Rene M Botnar, Tarique Hussain

**Affiliations:** 1 grid.452924.c0000000105407035Division of Imaging Sciences & Biomedical Engineering, British Heart Foundation Centre, King's College London, London, United Kingdom; 2 grid.267313.20000000094827121Department of Pediatric Cardiology, University of Texas Southwestern, Dallas, TX USA

## Background

Conventional 2 dimensional (2D) cine MRI of myocardial motion is limited in its ability to describe the complex 3 dimensional (3D) motion of the heart due to sparse coverage and inconsistent breath-holding. So far, clinical feasibility of 3D cine imaging has been limited due to the long acquisition times and requirements for acceleration techniques that are not routinely available.

We demonstrate the use navigator-gated 3D cine acquisition that can be performed with routine acceleration techniques within the time constraints of clinical MRI scans.

We compared the accuracy, reproducibility and image quality of 3D steady-state-free-precession (SSFP) cine imaging of the heart with conventional 2D SSFP cine imaging for the assessment of cardiac volumes, global and regional myocardial function.

## Methods

10 female subjects from the Twins UK cohort with no overt cardiac disease were prospectively recruited for a CMR study. Both conventional 2D short axis and 3D whole heart SSFP cine images were acquired in all ten cases. Patients with atrial fibrillation, valvular disease, regional wall motion abnormalities or areas of myocardial enhancement were excluded from the analysis. 3D scan parameters: FOV 320-400 mm; Acquired in-plane resolution 2 × 2 mm. Slice thickness 2 mm; b-FFE; parallel imaging acceleration 2; half-Fourier-factor 0.6; TR 3.7 / TE 1.87; flip angle 60; cardiac phases 25; scan duration 3 to 3.3 min, acquired in 10-12 breath-holds with navigator tracking. Volumetric and deformation analyses were done using semi-automatic contouring techniques with manual correction (Circle Cardiovascular Imaging v5.1.0).

Right (RV) and left (LV) ventricular volumes, global and regional strain and the LV systolic dyssynchrony index (LV-SDI) were compared for the two acquisitions. Intraclass correlation (ICC), Blant-Altman, inter- and intra-observer variability were analysed.

## Results

3D SSFP cine images showed good agreement with conventional 2D SSFP cine imaging for cardiac volumes (LV-EDV 2.3 ± 4.7 ml, *r*=.96, p<.01; LV-ESV -.58 ± 4.7 ml, *r* = .88 *p* < .01; LV-SV 3.1 ± 6.8 ml, *r*=.84 *p* = .01; RV-EDV 3.3 ± 7.9 ml, *r* = .92 *p* < .01; RV-ESV 0.8 ± 4.0 ml *r* = .89 *p* < .01; RV-SV -0.7 ± 4.2 ml, *r* = .96 *p* < .01), global circumferential strain (*r=*.85 *p* < .01) and global radial strain (*r* = .90 *p* < .01). The LV systolic dyssynchrony index is lower in the 3D acquisition when compared to the 2D SSFP images. Overall mean inter- and intra-observer correlation show better correlation for 3D than for 2D acquisition (2D *r* = .88 vs. 3D *r* = .93, *p* = .03). Image quality was no different between sequences (median IQ score 4.1 vs. 4.0, p = 0.59).

## Conclusions

Routine 3D SSFP cine imaging is feasible within clinical time constraints and without a loss of image quality or loss of agreement in absolute functional parameters compared to 2D measurements. More detailed coverage results in improved inter-observer agreement and reduced LV-SDI. The navigator-correction for the 3D approach also removed inaccuracies from slice mis-registration due to inconsistent breath-holding.

**Figure 1:**
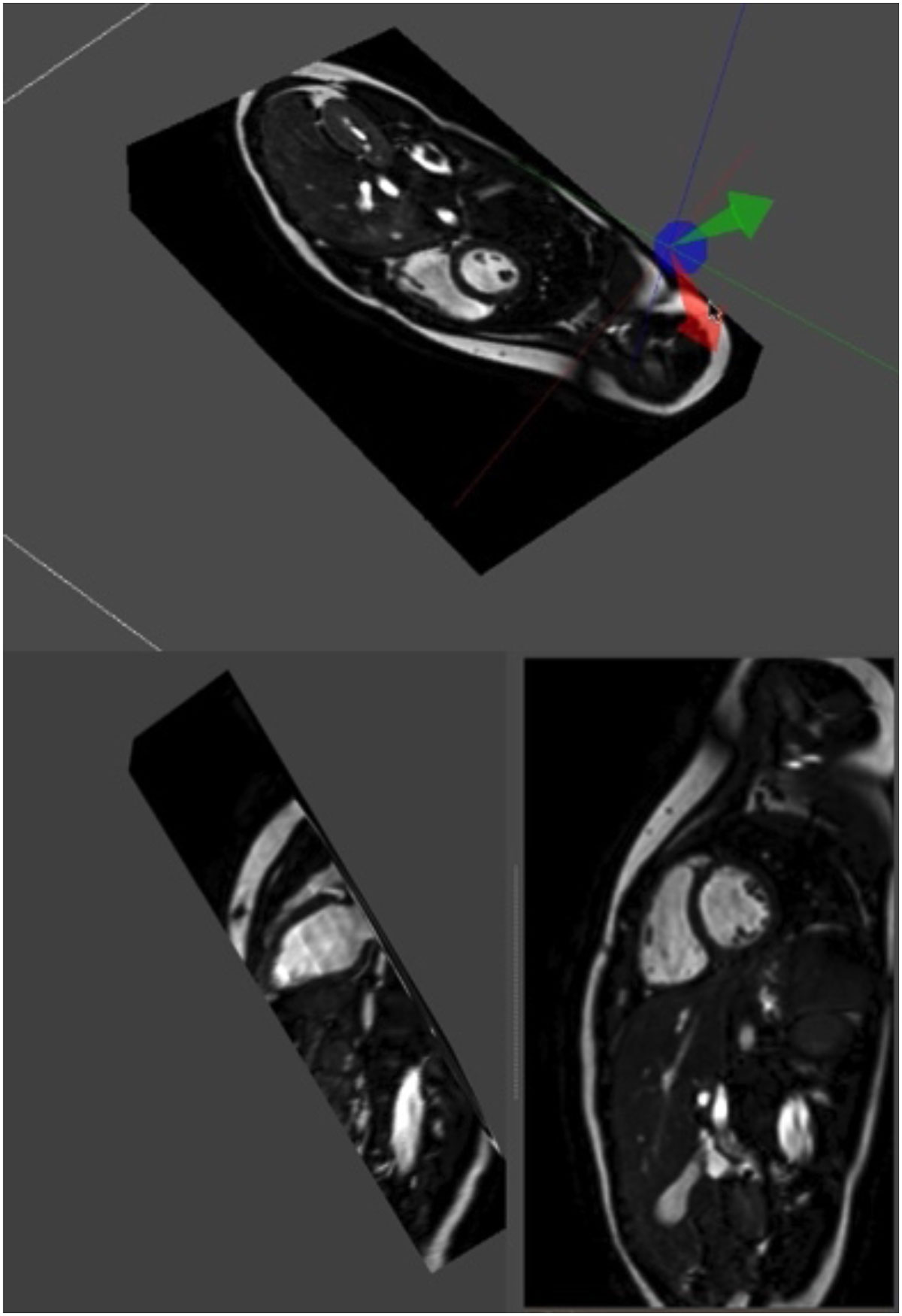
**3D balanced SSFP cine acquisition of the heart**. Using slice planes every image plane can be chosen. Top: 3D volume of the heart. Below: short axis and left ventricle long axis geometries are obtained from the original 3D volume, without inaccuracies from slice mis-registration.

**Figure 2:**
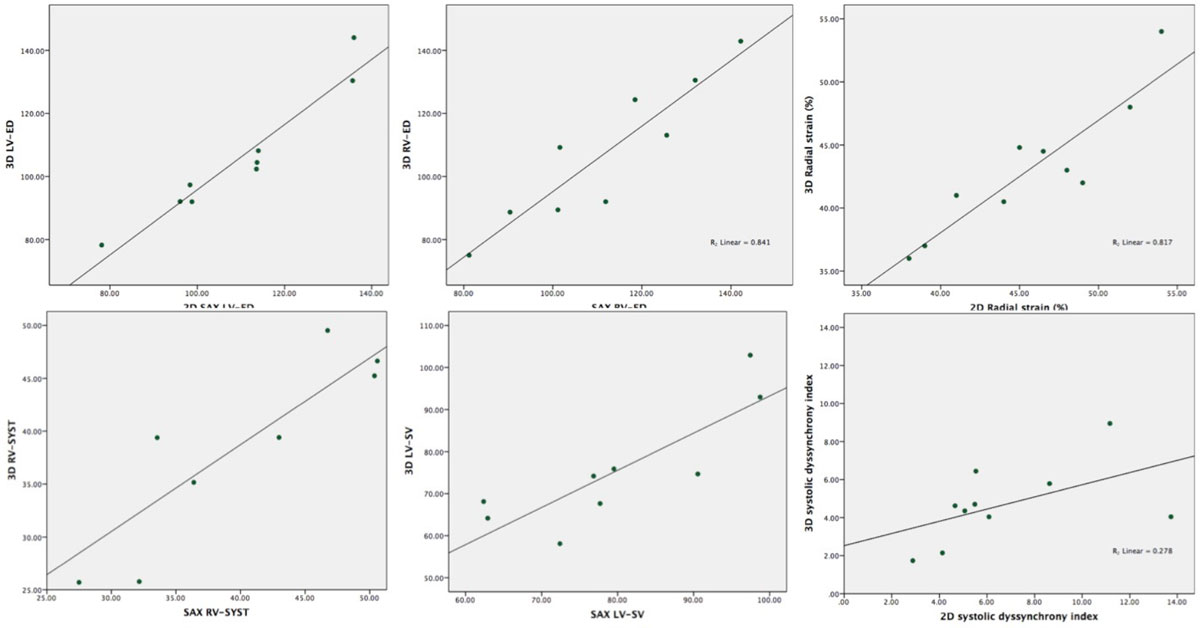
**A. Good correlation of left ventricular volumes between 2D and 3D acquisition (top; LV end-diastolic volume, below; LV end systolic volume)**. **B.** Good correlation of right ventricular volumes between 2D and 3D acquisition (top; RV end-diastolic volume, below; RV end systolic volume). **C.** Top; good correlation between global radial strain rates. Below; systematic lower dyssynchrony index in the 3D acquisition.

